# Differential Impact of Dietary Branched Chain and Aromatic Amino Acids on Chronic Kidney Disease Progression in Rats

**DOI:** 10.3389/fphys.2019.01460

**Published:** 2019-12-09

**Authors:** Samyuktha Muralidharan Pillai, Brigitte Herzog, Petra Seebeck, Giovanni Pellegrini, Eva Roth, François Verrey

**Affiliations:** ^1^Institute of Physiology and The Swiss National Centre of Competence in Research (NCCR) Kidney Control of Homeostasis (Kidney.CH), University of Zurich, Zurich, Switzerland; ^2^Institute of Physiology, University of Zurich, Zurich, Switzerland; ^3^Zurich Integrative Rodent Physiology (ZIRP), University of Zurich, Zurich, Switzerland; ^4^Laboratory for Animal Model Pathology (LAMP), Vetsuisse Faculty, Institute of Veterinary Pathology, University of Zurich, Zurich, Switzerland

**Keywords:** dietary proteins, amino acids, kidney function, chronic kidney disease, glomerular filtration rate, rat

## Abstract

The metabolism of dietary proteins generates waste products that are excreted by the kidney, in particular nitrogen-containing urea, uric acid, ammonia, creatinine, and other metabolites such as phosphates, sulfates, and protons. Kidney adaptation includes an increase in renal plasma flow (RPF) and glomerular filtration rate (GFR) and represents a burden for diseased kidneys increasing the progression rate of CKD. The present study aimed at identifying potential differences between amino acid (AA) groups constituting dietary proteins regarding their impact on RPF, GFR, and CKD progression. We utilized the well-established 5/6 nephrectomy (5/6 Nx) CKD model in rats and submitted the animals for 5 weeks to either the control diet (18% casein protein) or to diets containing 8% casein supplemented with 10% of a mix of free amino acids, representing all or only a subset of the amino acids contained in casein. Whereas the RPF and GFR measured in free moving animals remained stable during the course of the diet in rats receiving the control mix, these parameters decreased in animals receiving the branched chain amino acid (BCAA) supplementation and increased in the ones receiving the aromatic amino acids (AAAs). In animals receiving essential amino acids (EAAs) containing both BCAAs and AAAs, there was only a small increase in RPF. The kidneys of the 5/6 Nx rats receiving the BCAA diet showed the strongest increase in smooth muscle actin and collagen mRNA expression as a result of higher level of inflammation and fibrosis. These animals receiving BCAAs also showed an increase in plasma free fatty acids pointing to a problem at the level of energy metabolism. In contrast, the animals under AAA diet showed an activation of AMPK and STAT3. Taken together, our results demonstrate that subsets of EAAs contained in dietary proteins, specifically BCAAs and AAAs, exert contrasting effects on kidney functional parameters and CKD progression.

## Introduction

A high dietary protein load increases both renal plasma flow (RPF) and glomerular filtration rate (GFR) acutely and in the long term, and increases renal expression of proinflammatory genes (Hostetter et al., 1986; Tovar-Palacio et al., 2011; Juraschek et al., 2013). In the case of Dahl salt-sensitive rats, high protein diet has been shown to increase immune cell infiltration of kidneys, blood pressure, and kidney damage (De Miguel et al., 2011). As early as 1948, a restriction of protein intake has been suggested to prevent the increase in “workload” of surviving nephrons in patients with chronic kidney disease (CKD) (Brenner et al., 1982). Importantly, recent studies confirm the utility of restricting protein intake in CKD patients to slow down CKD progression (Koppe and Fouque, 2019).

Interestingly, not all proteins equally induce hyperfiltration and some of their constituent AAs, for instance, branched chain amino acids (BCAAs) appear to be less effective than others (Claris-Appiani et al., 1988). There is however a lack of recent studies investigating underlying mechanisms (Alvestrand and Bergstrom, 1984; Friedlander et al., 1990; Luippold et al., 2004, 2006). Nonetheless, it appears crucial that renal adaptation to protein intake, including hyperfiltration, is necessary for the excretion of the nitrogenous end products (urea, ammonia, etc.) and other protein-associated wastes including acid equivalents due to the metabolism of sulfur-containing and cationic amino acids. This adaptation involves the combined effects of the hormones vasopressin and glucagon that together contribute to efficient urea excretion and water economy (Bankir et al., 2015, 2018). Furthermore, handling of the acid load from proteins of animal origin is considered to contribute to accelerated CKD progression (Scialla and Anderson, 2013).

Besides the smaller effect of BCAAs on hyperfiltration and the potentially more harmful effect of sulfur-containing and cationic amino acids due to acid production, it is not known whether specific amino acid groups may differentially affect kidney function and CKD progression.

To test the potential differential effects of various amino acid groups on CKD progression, we chose to use the well-established 5/6 nephrectomy (5/6 Nx) model that corresponds to the situation of a reduced number of nephrons in the absence of specific glomerular or tubular lesions (Lim et al., 2014). To assess the kidney function repeatedly in these rats in awake condition, we measured the GFR using the transcutaneous FITC-sinistrin concentration-decay curve method (Scarfe et al., 2018). For measuring renal plasma flow, we developed a technique that allowed us to measure the clearance of exogenous PAH applied while rats were awake. This was however only possible to perform at the end of the experiment. Practically, radioactive PAH was released by an osmotic mini pump and its urinary excretion measured over 24 h in a metabolic cage. Its renal extraction from arterial blood was then determined by measuring its concentration in arterial and renal venous blood at the end of the urine collection, after sacrificing the rat.

The diets enriched in different amino acid groups given to the rats were based on the usual laboratory rat diet (AIN93) and contained all 18% caloric content as protein and amino acids. Eight percent (minimal dietary protein caloric fraction for rats; Meireles et al., 1999) of these was given as casein and the other 10% as free amino acids. These amino acid mixes had the same relative concentration as in casein, either of all amino acids (8 + 10), or only of a subset of them, namely the non-essential ones (NEAAs), the essential ones (EAAs), the branched chain ones (BCAAs), or the aromatic ones (AAAs). Submitting 5/6 Nx rats to these diets, we could demonstrate that the amino acid composition of the proteinaceous part of the diet differentially affects CKD progression.

## Methods

### Animals

Experiments were performed with male 250- to 300-g (10–12 weeks old) Wistar Han rats purchased from Charles River (Germany). All procedures for rat handling and experimental interventions were performed in accordance with Swiss Animal Welfare laws and approved by the Cantonal Veterinary Office, Zürich. Rats were group-housed in standard laboratory conditions with a 12-h light/dark cycle. They had free access to food and water.

### Diets

Rats were fed with the maintenance diet containing 18% protein (2222 Granovit, Switzerland) during acclimatization and 4 weeks after the surgery. At week 4, GFR was measured and the animals were then shifted for the end of the experiment to modified diets as described below and on which they remained for the rest of the experiment. The modified diets (variants of 2222, Granovit, Switzerland) were designed to contain 8% protein and 10% amino acid mix in the same proportion as that found in casein (Supplementary Table S4). Thus, for all diets, the AAs not part of the AA mix (only present in the 8% casein) were present at 44.5% of their level in an 18% casein diet and in the 8 + 10 diet. In the case of the NEAA diet (11 non-essential amino acids in the 10% AA mix: Ala, Arg, Asn, Asp, Cys, Gln, Glu, Gly, Pro, Ser, Tyr), each of the 11 NEAAS was present in total (in casein + AA mix) at a level representing 146% of what the pure casein diet (18% of calories) and the 8 + 10 diet contained. In the case of the EAA diet (nine EAAs: His, Ile, Leu, Lys, Met, Phe, Thr, Trp, Val), their total concentration was 167% of the concentration present in the casein and the 8 + 10 diets. In the case of the BCAA and the AAA diets (each only three AAs; BCAAs: Ile, Leu, Val; AAAs: Phe, Trp, Tyr), the proportion of these AAs was 450% of their concentration in the 18% casein or the 8 + 10 diets. The study is based on four experimental series and the results of identical groups have been pooled. Due to the lack of change in our readouts, some dietary groups were not repeated. Therefore and because not all measurements have been performed in all experimental series, the numbers vary between the different groups and determinations.

### Surgeries

The experiments were started after at least 1 week of acclimatization. For the surgery, rats were shortly anesthetized with isoflurane. The UNX was performed first on the right kidney and the 2/3 Nx (second surgery) was performed a week later on the left kidney. The area undergoing the surgery was shaved the day before, in case of both operations. For the UNX, an incision was made on the right side. The adrenal was carefully separated from the upper pole of the kidney before ligating the renal pedicle. The right kidney was then removed and the peritoneum and the skin sutured. The animals were allowed to recover for 1 week prior to performing the 2/3 Nx. Under isoflurane anesthesia, the left kidney was exposed and the adrenal separated before the two poles of the kidney were amputated and covered with gel foam (Spongostan, Ethicon, USA) as described (Van Koppen et al., 2013).

For sham surgeries, the same process was followed on both the left and right sides with a 1-week gap between surgeries. In both cases, the kidneys were exposed but not ablated.

### Glomerular Filtration Rate Measurements

Rats were shaved the evening prior to measurement. On the day of the measurement, the rats were briefly anesthetized with 1.5% isoflurane and the transcutaneous GFR measurement camera (Medibeacon, Germany) was attached *via* a sticky patch and surgical tape to the rat. FITC-sinistrin (Fresenius Kabi, Germany) at a concentration of 7 mg/100 g body weight was injected *via* the tail vein. The rat was allowed to wake up and placed alone in a cage for 2 h. Following this, the camera was removed from the rat, the rat returned to the home cage, and the measurement from the camera analyzed using MPD studio (Medibeacon, Germany).

### Renal Plasma Flow Measurements

RPF measurements were performed as terminal experiments in most animals in which GFR had been previously tested. An osmotic pump (2ML1 Charles River, Germany) containing a solution of [^3^H] PAH (Perkin Elmer, USA) and 10 μM unlabeled PAH (with HEPES as a buffer) in saline was implanted into the rat that was put into the metabolic cage for 24 h. The following day, food was taken away for 1 h before the animal was anesthetized (3% isoflurane), and blood was collected from both the renal vein and the aorta for RPF calculations. The urine collected in the metabolic cage provided the information for urinary flow rate and urinary tracer measurements. Tubes were prepared containing either 100 μl of plasma or 100 μl of urine. A volume of 3 ml of ultimate GoldTM scintillation fluid (Perkin Elmer, Waltham, MA, USA) was added and the tubes were shaken for 2 h following which the level of radioactivity was measured using the liquid scintillation analyzer (Packard Tri-Carb 2900TR, PerkinElmer, USA). The RPF was then calculated by using the formula RPF (ml/min) = (U*V)/(Pa − Pv) where U is the urinary concentration of [^3^H] PAH, V is the urinary flow rate in ml/min, Pa is the arterial plasma concentration of [^3^H] PAH, and Pv is the venous plasma concentration of [^3^H] PAH.

### Body Composition Measurements

Measurements were performed using the ECHO-MRI (ECHO Medical Systems, USA). Calibrations and measurements were performed according to manufacturer’s instructions.

### Ultra-Performance Liquid Chromatography Amino Acid Measurements

Amino acid concentration analysis was performed at the Functional Genomic Centre Zurich (FGCZ), using the Mass Track Amino Acid Analysis Application Solution by ACQUITY ultra-performance liquid chromatograph (UPLC; Waters Corporation, Milford MA, USA) according to the manufacturer’s instructions. Plasma samples were diluted to 1:1 with 10% sulfosalicylic acid for deproteinization prior to UPLC.

### Measurement of Free Fatty Acids, Creatinine, and Electrolytes

FFA were measured using the ASC-ACOD Method (a colorimetric assay) following the manufacturer’s instructions (Fujifilm Wako, Germany). Measurements of sodium, potassium, magnesium, chloride, calcium, phosphorous, urea, and creatinine were done on UniCel DxC 800 Synchron Clinical System (Beckman Coulter), a service provided by the Zürich Integrative Rodent Physiology (ZIRP) facility following the manufacturer’s instructions.

### Quantitative Real-Time Polymerase Chain Reaction

Tissue samples were lysed using Trizol (Ambion, Thermo Fisher Waltham, MA, USA) with a Precellys homogenizer (Bertin instruments, Montigny-le-Bretonneux, France). Total RNA was extracted using the RNeasy mini kit (Qiagen, Hilden, Germany) according to the manufacturer’s instructions. RNA concentration was determined using a Nanodrop (Agilent Technologies, Santa Clara, CA, USA) and 500 ng was used to synthesize single-strand cDNA in 20 μl reactions using the qScript cDNA Synthesis Kit (Quanta Biosciences, Beverly, MA, USA). The values were expressed relative to GAPDH (2^∆Ct^).

Quantitative polymerase chain reaction (qPCR) primers were either based on previously reported sequences (Ding et al., 2012; Dizin et al., 2013) or designed using the NCBI primer blast tool.^1^ The primers used were as follows: GAPDH forward 5′-GCAAGAGAGAGGCCCTCAG-3′ and GAPDH reverse 5′-TGTGAGGGAGATGCTCAGTG-3′, α-SMA forward 5′-ACTGAGCGTGGCTATTCCTT-3′ and reverse 5′-TTCTCCAGGGAAGAAGAGGA-3′, COL1A1 forward 5′-ATCTCCTGGTGCTGATGGAC-3′ and reverse 5′-ACCTTGTTTGCCAGGTTCAC-3′, COL1A2 forward 5′-CCCCAAAACGTTTGCAGTGT-3′ and reverse 5′-GACAATGTCCACAACAGGTGTC-3′, COL3A1 forward 5′-CCCCAAAACGTTTGCAGTGT-3 and reverse 5′-GACAATGTCCACAACAGGTGTC-3′. qPCR was performed in a 96-well format and consisted of 2 μl of diluted cDNA per well, PowerUp SYBR Green Mastermix (Applied Biosystems, Foster City, CA, USA) and 0.5 μM of each primer in a 20 μl reaction. Thermal cycling conditions were an initial denaturation step of 94°C for 2 min, and then 40 cycles of 94°C for 15 s and 57°C for 1 min. Each experimental cDNA was measured in triplicate.

### Western Blot

Samples were homogenized with beads (Roche, cat#03358941001) at 6,000 rpm for 30 s in a mannitol-based resuspension buffer (200 mM D-Mannitol, 80 mM HEPES, 41 mM KOH, pH 7.5, 0.8% NP40) supplemented with protease and phosphatase inhibitor cocktails in a Precellys 24 device (France). The homogenate was then centrifuged at 2,000 *g* for 15 min at 4°C to sediment cellular debris. Supernatant was ultracentrifuged at 41,000 rpm for 10 min at 4°C (rotor RP45A, Sorvall, ThermoFisher Scientific, Waltham, MA, USA) to eliminate small debris. Protein concentration was determined using Pierce™ BCA Protein Assay (Cat#23228, Lot#UA269551, ThermoFischer Scientific, Rockford, IL, USA) according to the manufacturer’s instructions.

Phospho and total AMPK, phospho and total STAT3, α-SMA, β-tubulin antibodies used for western blotting were purchased from CST (USA). The blots were performed according to manufacturer’s instructions. When anti-phospho and total protein antibodies were used, first the phospho proteins were imaged using the corresponding antibody and HRP staining. Then, the membrane was stripped using the Abcam (Germany) harsh stripping protocol that includes β-mercaptoethanol. Finally, the total proteins were visualized on the same blot by using AP (Millipore, Switzerland). Chemiluminescence was detected with a LAS-4000 camera system (Fujifilm). Densitometric analysis was performed using ImageJ.

### Microscopy, Immunohistochemistry, and Quantification

Remnant left kidneys that had undergone partial nephrectomy were removed, sliced, and fixed in 10% neutral-buffered formalin (Formafox, Hittmau, Switzerland), followed by dehydration through graded alcohols and embedding in paraffin wax. Consecutive sections (3–5 μm) were prepared, mounted on glass slides, and subjected to hematoxylin and eosin (HE) staining. Immunohistochemistry for the detection of alpha smooth muscle actin (α SMA) was carried out on the renal sections using an antibody against alpha-smooth muscle actin (clone 1A4; DAKO, Basel, Switzerland) in a Dako Autostainer (Dako Autostainer Universal Staining System Model LV-1, Dako-Agilent Technologies). All slides were scanned using a digital slide scanner (NanoZoomer-XR C12000; Hamamatsu, Japan) and histomorphometrical analysis was performed on the digital slides using the Visiopharm Integrator System (VIS, version 4.5.1.324, Visiopharm, Hørsholm, Denmark). α-SMA-positive areas were quantified in each animal using at least 40 fields per kidney section, avoiding regions containing large α-SMA-positive vessels. Results were expressed as average fraction of α-SMA-positive areas normalized against the total renal parenchyma.

### Statistical Analysis

GraphPad Prism software (version 6; GraphPad Software Inc., San Diego, CA) was used to perform statistical analysis. Differences between groups were determined by Student’s *t*-test for two groups (paired in case the same animal was being followed over time) or one-way repeated-measures ANOVA for ≥3 groups with a Dunnett *post hoc* test versus a control group. Values are presented as means ± SEM and were considered different when *p* < 0.05.

## Results

**Figure 1:**
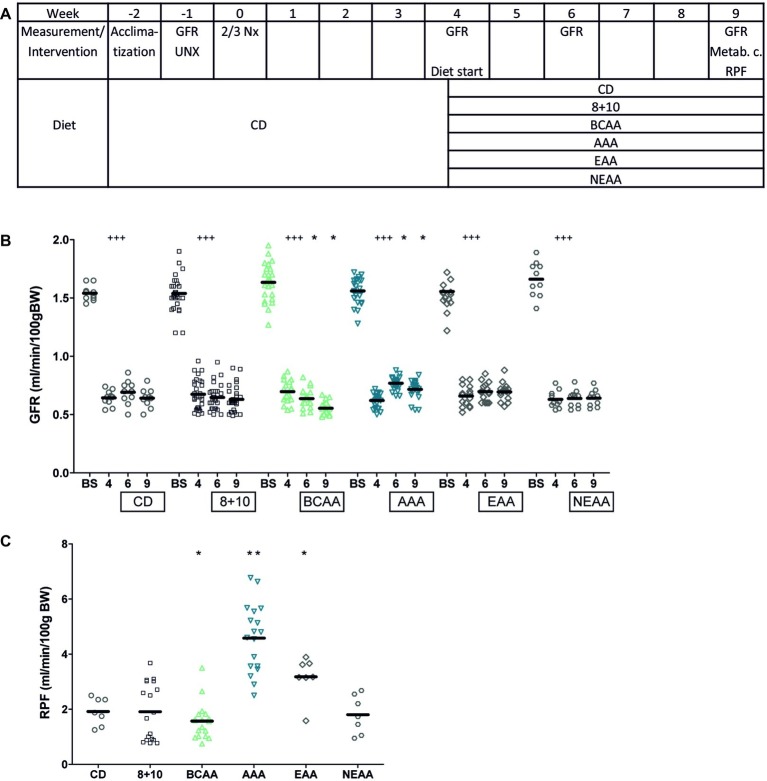
Impact of amino acid diets on GFR and RPF. **(A)** Schematic representation of experimental plan. CD stands for control diet (18% casein), 8 + 10 for 8% casein +10% casein amino acid mix, BCAA, AAA, EAA, and NEAA for 8% casein +10% of branched chain, aromatic, essential, and non-essential amino acid mix, respectively. **(B)** Impact of 5/6 nephrectomy (5/6 Nx) on GFR measured in free moving animals. The GFR was measured a first time before first surgery (uninephrectomy) (BS) and again 4 weeks after the second surgery just before starting the diet (4). The animals were then given different diets as indicated in the boxes below the *X* axis. GFR was measured again 2 and 5 weeks after the start of the diet (weeks 6 and 9). 5/6 Nx animals under BCAA diet showed a further decrease in GFR (green) and those under AAA diet an improvement (blue) compared to values at week 4. ^###^*p* < 0.005 (comparing values at week 4 to those before surgery with a paired *t* test), *p < 0.05 (ANOVA with a Dunnett *post hoc* test). CD, *n* = 10; 8 + 10, *n* = 30; BCAA, *n* = 25; AAA, *n* = 25; EAA, *n* = 15; and NEAA, *n* = 10. **(C)** Impact of different diets on RPF in CKD animals (5/6 Nx). RPF was measured 5 weeks after starting the diet (week 9). The BCAA group (green) showed a decrease in RPF, whereas both the EAA and the AAA (blue) groups showed an increase compared to the 8 + 10 group (black). *p < 0.05; **p < 0.01; CD, *n* = 7; 8 + 10, *n* = 18; BCAA, *n* = 18; AAA, *n* = 18; EAA, *n* = 7; and NEAA, *n* = 7.

To test whether the amino acid composition of dietary proteins was important in the progression of CKD, we designed an experimental protocol using 5/6 nephrectomy (5/6 Nx) as CKD model in rats. After 6 weeks on normal control diet (CD) containing 18% casein (Figure 1A), we submitted these animals to different isocaloric diets containing 8% protein, supplemented with 10% of an amino acid mix containing one of the following: all amino acids as in casein (8 + 10), branched chain amino acids (BCAAs), aromatic amino acids (AAAs), essential amino acids (EAAs), or non-essential amino acids (NEAAs). Using the FITC-sinistrin clearance technique, GFR was measured before the first surgery and again in 5/6 Nx (CKD model) animals 4 weeks after the second surgery (Figure 1A). The second measurement showed that GFR had decreased as expected by more than 50%, namely from 1.5 ± 0.06 ml/min/100 g BW before surgery to 0.6 ± 0.04 ml/min/100 g BW after 5/6 Nx (Figure 1B, *p* < 0.005). The animals were then randomly assigned to the different diet groups and the GFR was measured again after 2 and 5 weeks of diet (at weeks 6 and 9) (Figure 1B). Interestingly only the BCAA group showed a significant decrease in GFR over this period, namely from 0.7 ± 0.03 ml/min/100 g BW at week 4 to 0.52 ± 0.02 ml/min/100 g BW at week 9 (*p* < 0.05). In contrast, the AAA group showed the opposite effect with GFR increasing from 0.63 ± 0.03 ml/min/100 g BW at week 4 to 0.72 ± 0.05 ml/min/100 g BW at week 9 (*p* < 0.05).

RPF was measured in rats 9 weeks post second surgery. Similar to GFR, we observed a reduction in the case of BCAA diet (1.55 ± 0.26 ml/min/100 g BW) (Figure 1C) compared with the 8 + 10 group (1.93 ± 0.25 ml/min/100 g BW, *p* < 0.05) and a strong increase in RPF for the AAA diet group (4.61 ± 0.45 ml/min/100 g BW, *p* < 0.01) and to a lesser extent for the EAA group (3.17 ± 0.21 ml/min/100 g BW, *p* < 0.05). Interestingly, the EAAs that contained both the AAAs and the BCAAs produced an effect resembling more that of AAAs. There were no changes in the GFR and RPF of sham-operated animals on different diets (Supplementary Tables S1, S2). Additionally, no significant changes were observed between animal groups in terms of body weight, food and water consumption, urinary output and proteinuria. The fractional excretion of the tested electrolytes was comparable across the different diets with the exception of phosphorous, which was decreased in the BCAA group (Table 1).

**Table 1:** Effect of amino acid supplementation on general parameters and fractional excretion (FE) of electrolytes and urea.

	8 + 10		BCAA		AAA	
	Mean	SEM	Mean	SEM	Mean	SEM
Body weight (g)	345.6	7.3	336.8	11.9	342.0	11.5
Food/24 h (g)	14.7	1.5	15.6	1.2	15.2	2.5
Water/24 h (g)	35.3	6.0	30.6	1.9	27.9	2.3
Urine/24 h (ml)	14.6	2.2	14.4	0.8	12.4	2.2
Proteinuria/24 h (mg)	52.2	4.1	54.1	7.9	54.1	7.0
Feces/24 h (g)	1.4	0.6	1.0	0.6	1.0	0.2
	**Fractional excretion (%)**					
Na^+^	0.2	0.0	0.1	0.0	0.2	0.0
K^+^	14.6	1.0	11.0	1.3	12.9	1.7
Cl^−^	0.6	0.0	0.5	0.1	0.4	0.0
Ca^2+^	0.7	0.1	0.7	0.1	0.4	0.1
Mg^2+^	6.8	2.1	5.0	1.1	8.9	1.8
Pi	5.1	0.8	1.4*	0.8	5.7	1.8
Urea	57.0	2.1	50.7	2.8	53.3	4.5

* *p < 0.05, compared to 8 + 10 CKD group, *n* = 10 animals per group*.

**Figure 2:**
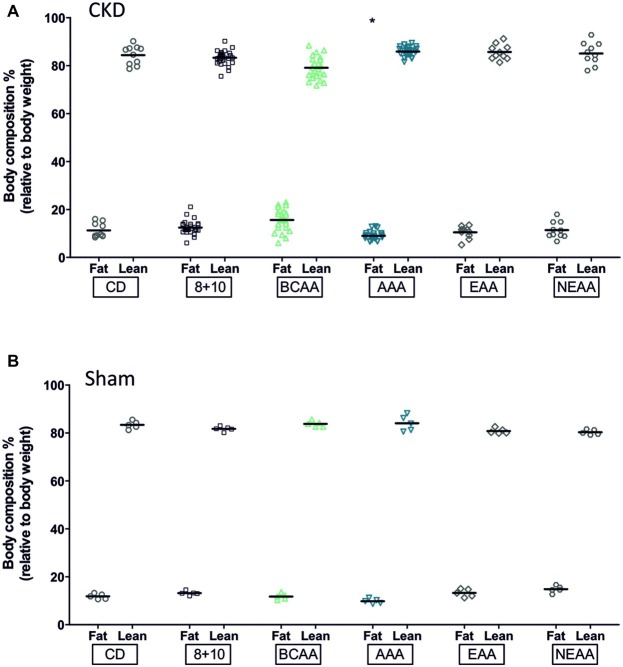
Effect of amino acid supplementation on body composition of CKD and Sham animals. CKD and Sham animals on different diets did not show changes in fat (black bars) and lean mass (gray bars for CKD animals and white for controls) with the exception of the AAA groups. They showed a reduction in fat mass that was significant in CKD (*p < 0.05) and with a *p* slightly above the 0.05 limit for the sham group, *p* = 0.06, ANOVA with Dunnett *post hoc* test. CKD: CD, *n* = 10; 8 + 10, *n* = 30; BCAA, *n* = 25; AAA, *n* = 25; EAA, *n* = 15; and NEAA, *n* = 10. Sham: *n* = 5 animals per group.

We also evaluated the body composition of the different animal groups using ECHO MRI (Figure 2). The CKD-AAA animals showed a significant reduction in fat (*p* < 0.05) whereas the sham-operated AAA animals also showed a reduction in fat, albeit not statistically significant. This suggests that the fat decrease was induced by the AAA diet, with little influence of CKD. Note that the BCAA-CKD group showed a non-significant increase in fat (*p* = 0.06). This effect was observed only in the CKD group and not in the sham-operated animals.

**Figure 3:**
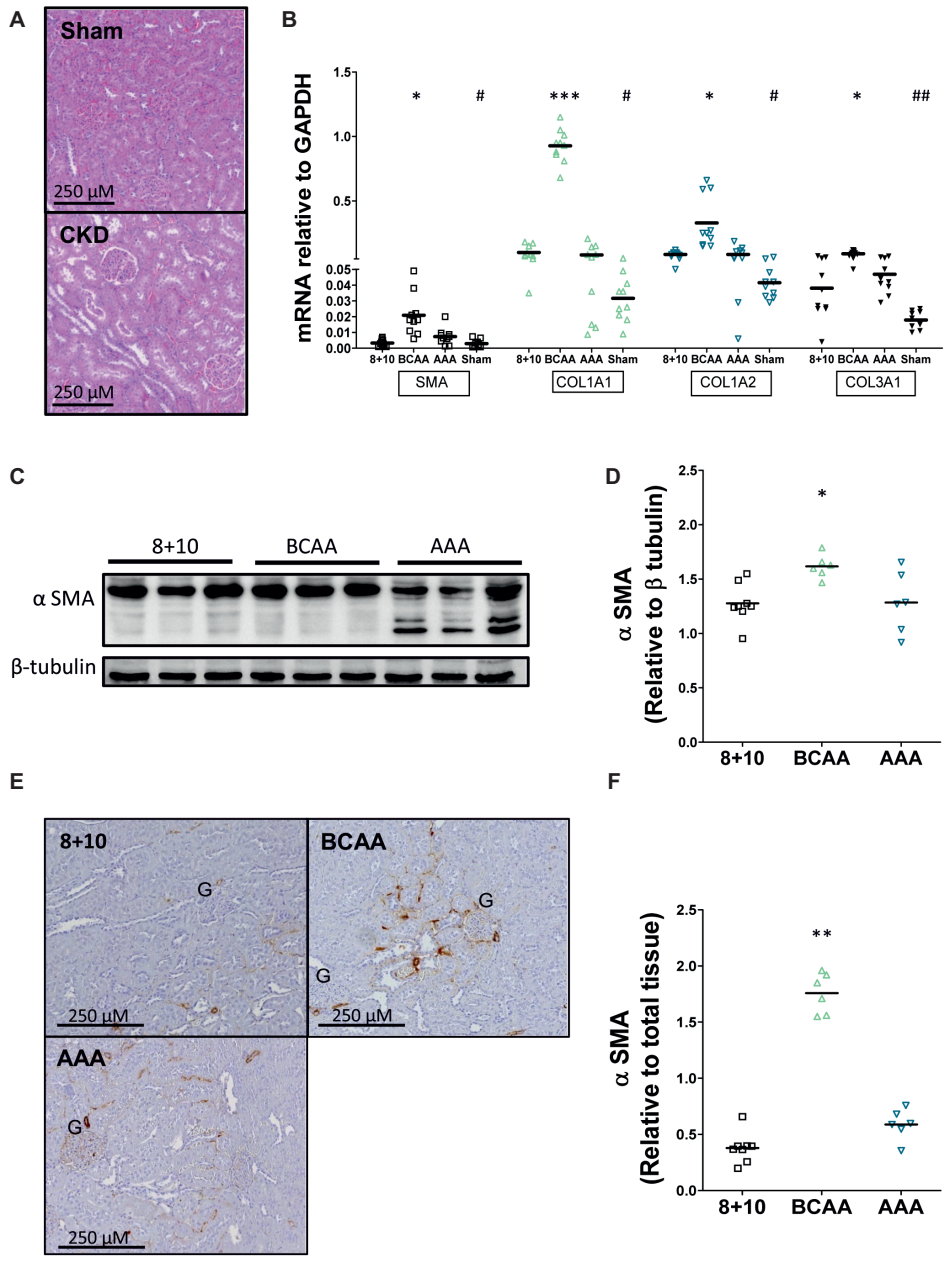
Impact of amino acid diets on fibrosis markers. **(A)** Hematoxylin-eosin labeled kidney sections of 5/6 Nx and sham-operated rats. Kidneys of 5/6 Nx animals show hallmarks of CKD including glomerular hypertrophy and mesangial matrix expansion, unlike sham-operated animals. **(B)** mRNA of α SMA and collagen in CKD and sham-operated animals on different diets. A significant increase in α SMA, COL1A1, COL1A2, and COL3A1 mRNAs was observed in CKD versus sham-operated animals on 8 + 10 diet. ^#^*p* < 0.05, ^##^*p* < 0.05. Among CKD animals, there was no difference between AAA and 8 + 10 diets, whereas all mRNAs were significantly increased under BCAA diet. *p < 0.05, ***p < 0.001, ANOVA with Dunnett *post hoc* test *n* = 10. **(C–F)** Protein levels of α SMA measured by western blot **(C,D)** and visualized by microscopy **(E,F)**. α SMA levels were increased only in the BCAA group. The reason for α SMA fragmentation seen on western blot specifically in AAA samples is not known. Representative blots and sections are shown. *p < 0.05, **p < 0.01, ANOVA with Dunnett *post hoc* test. 8 + 10, *n* = 8, BCAA and AAA, *n* = 6.

We investigated the impact of the different diets on kidney morphology and fibrosis first by visualizing its structure on hematoxylin and eosin (HE)-stained sections (Figure 3A). Kidney sections from 5/6 Nx animals exhibited glomerular hypertrophy with mesangial matrix expansion, consistent with CKD injury described in this model. However, no notable differences were observed between the different dietary groups. We then specifically evaluated α SMA and collagen levels, since they represent reliable indicators of the progression of CKD. The mRNA expression level of both α SMA and collagen (collagen 1A1, 1A2, and 3A1) was increased at least two-fold in CKD animals relative to sham-operated ones (on 8 + 10 diet) (Figure 3B). Within the CKD animals, the BCAA group showed, compared with the 8 + 10 group, a significant increase in the expression of all three tested collagens [1A1 (*p* < 0.005), 1A2 (*p* < 0.05), and 3A1 (*p* < 0.05)] as well as α SMA (*p* < 0.05) compared with the 8 + 10 group.

To study the expression of α SMA at the protein level, we performed both western blots (Figures 3C,D) and quantitative immunohistochemical analysis (Figures 3E,F). Both methods also showed significantly increased levels of α SMA only in the BCAA group relative to the 8 + 10 one (*p* < 0.05 and <0.01, respectively).

Furthermore, we tested the plasma amino acid concentration, which has been shown by others to vary depending on the progression of CKD (Duranton et al., 2014). Interestingly, the BCAAs and AAAs that were highly enriched in the corresponding diets, tended to be lower in the plasma of the respective groups as measured during the resting phase (1 h after food removal). However, these potential changes were not statistically significant compared to the 8 + 10 control group (Supplementary Table S3).

**Figure 4:**
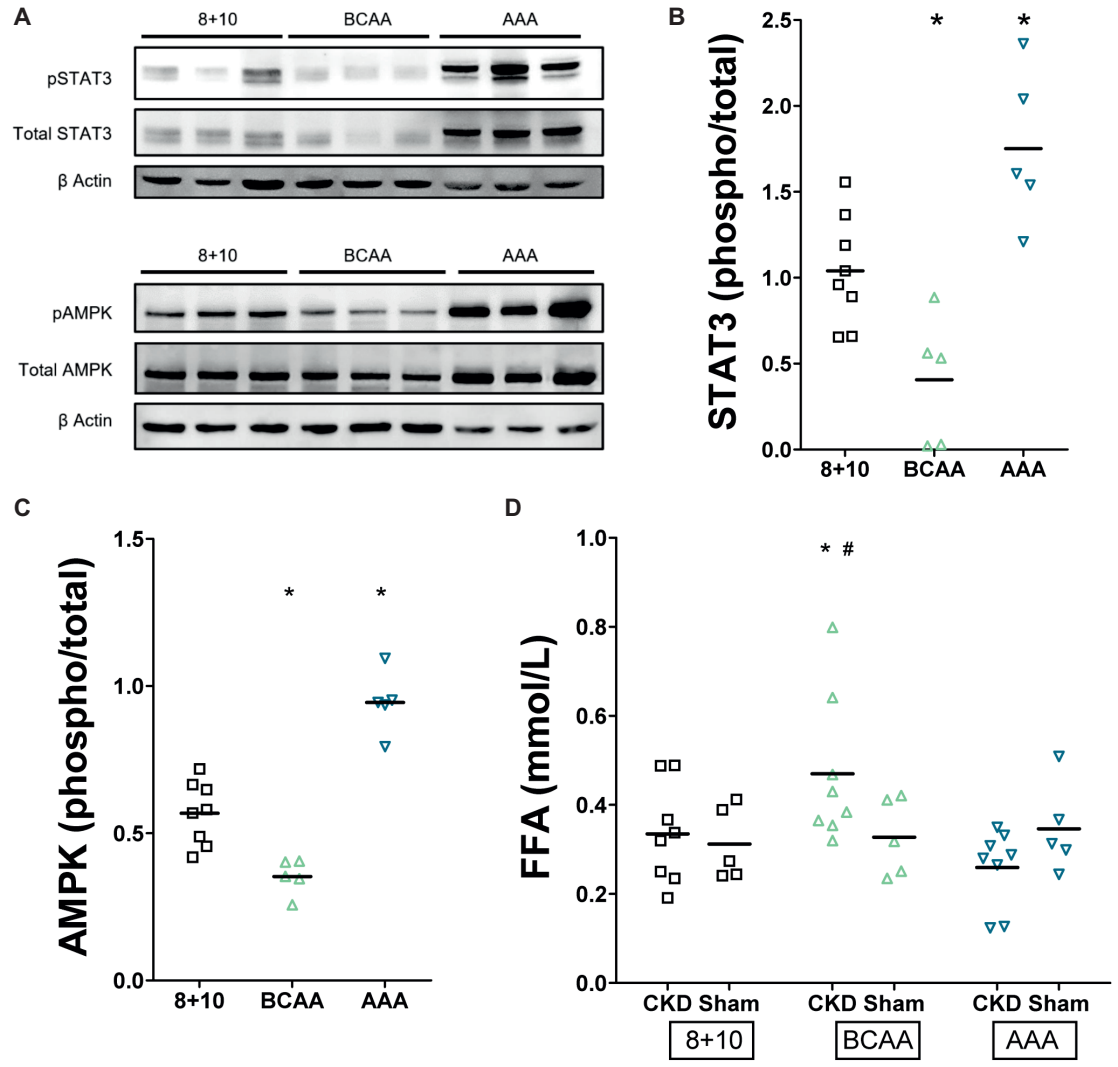
Impact of CKD and amino acid diets on renal pSTAT3 and pAMPK and on plasma free fatty acids (FFA). **(A)** Western blot. Total and phosphorylated STAT3 appear as two bands of approximately 79 and 86 kD and AMPK as one band around 62 kDa. Both are possibly decreased under BCAA diet and clearly increased under AAA diet. **(B)** Quantitation of pSTAT3/STAT3 phosphorylation ratio (*n* = 5), *p < 0.05 compared to 8 + 10 animals. **(C)** Quantitation of pAMPK/AMPK phosphorylation ratio (*n* = 5), *p < 0.05 compared to 8 + 10 animals. **(D)** Plasma free fatty acids. There is no difference between CKD and control animals under 8 + 10 and AAA diets. The FFA are, however, elevated in CKD animals under BCAA diet. *^,#^*p* < 0.05 comparing to CKD and Sham animals, respectively, both under 8 + 10 diet (ANOVA with Dunnett *post hoc* test). Sham, *n* = 5; CKD, *n* = 8 animals per group.

Recent studies have identified markers of tubular overload and attempted to elucidate the mechanisms by which such functional overload and consecutive damage lead to interstitial fibrosis as observed in CKD. One of the important signaling pathways activated by functional overload is that of AMP-activated protein kinase (AMPK). Interestingly, this pathway was repressed in the remnant kidney of CKD animals under BCAA diet (decreased phospho−/total AMPK ratio) whereas it was activated in that of the AAA diet group (increased phospho−/total AMPK ratio) (Figures 4A,C, *p* < 0.05). STAT3 has emerged as a major mediator of tubulointerstitial cross talk in CKD (Bienaime et al., 2016). Parallel to the situation with AMPK, we noted in our experiments that phospho/total STAT3 was reduced in kidneys of CKD animals under BCAA diet, whereas it was increased in the kidney of rats under AAA diet compared to the 8 + 10 group (Figures 4A,B). An intriguing observation is the apparent increase in total STAT3 and AMPK protein under AAA diet that is well visible in comparison with beta-actin (Figure 4A). This apparent increase in AMPK and STAT3 protein expression level adds up to the increased proportion of their phosphorylated forms and might point to an additional mechanism by which the AAA dietary load acts.

We measured plasma free fatty acids because of the rapid deleterious effect of the BCAA diet in CKD and since branched chain amino acid metabolism is known to feed into β-oxidation and citric acid cycle and thus to compete with fatty acid metabolism (Newgard, 2012). We found their level was indeed elevated specifically in the CKD group with BCAA diet (Figure 4D, *p* < 0.05).

## Discussion

This study shows that the administration of dietary proteins containing different proportions of specific amino acid groups differentially impacts on CKD progression. Differences between the groups became visible already 2 weeks after initiation of the diet. In particular, the BCAA group showed a further decrease in GFR and the AAA group an increase. These changes in GFR were parallel to changes in RPF. Surprisingly, the EAA group receiving a diet containing an increased proportion of both BCAAs and AAAs also showed an increase in RPF, more similar to the AAA than the BCAA group.

The AAA group rats had a smoother body texture and interestingly this difference correlated with a lower proportion of fat measured by ECHO MRI (Figure 2). However, there were no other gross morphological differences observed between sham-operated rats and the different CKD groups. At the level of the kidneys, all CKD animals typically showed glomerular hypertrophy, mesangial expansion, and increased markers of fibrosis such as α SMA and collagen (Figures 3A,B; Gava et al., 2012; Lim et al., 2014). The BCAA diet group showed, compared to the CKD 8 + 10 and the AAA groups, a significant further increase in both fibrosis markers (Figures 3B–F). In a search for possible mediators, we tested tubular STAT3 that has recently emerged as an important mediator of the tubulointerstitial cross talk that leads to interstitial fibrosis in CKD (Bienaime et al., 2016). Surprisingly, the kidney of the more affected BCAA group showed a decrease in phospho/total STAT3 ratio whereas the apparently protected AAA group showed an increase. A possible explanation is that the BCAA animals had already passed the stage of elevated pSTAT3 expression at the time of the measurement (5-week diet), whereas the AAA group had first developed an increased plasma flow and GFR and correspondingly experienced an additional functional overload leading to an activation of the AMPK pathway. It may be that the activation of this pathway, which lies at the crossroads of energy metabolism, ion transport, and inflammation, has exerted a protective effect in the kidney of the AAA group (Rajani et al., 2017). However, it has not been established why the AAA-rich diet affected the RPF and correspondingly the GFR specifically in CKD conditions. It has to be mentioned that the GFR increase was proportionally smaller than that of the RPF, indicating a decrease in fractional filtration and suggesting a dilation of both vasa afferentia and efferentia. We might speculate that the high amount of dietary aromatic amino acids led to an increase in the production of (vasoactive) catecholamines and/or tryptophan derivatives in the AAA group. These mediators could have led not only to the decrease in fat observed also in sham-operated animals, but also to an increase in RPF specifically in the 5/6 Nx CKD model in which the tubuloglomerular feedback is known to be inoperative (Singh et al., 2012). Thus, after 5 weeks of high-aromatic amino acid diet, the functional strain due to increased RPF and GFR presumably started to cause deleterious effects as suggested by the elevated expression and activation of STAT3. This effect is in agreement with earlier observations showing that a long-term exposure to a high-protein diet causes an increase in GFR but that this hyperfiltration may have over time deleterious effects (Brenner et al., 1982; Koppe and Fouque, 2019).

The question of the mechanism leading to the much more rapid alteration of the remnant kidney under BCAA diet remains to be elucidated. A hint toward a role of metabolic imbalance comes from the observation that plasma free fatty acids were elevated and phosphorylated AMPK decreased in the BCAA group (Figures 4C,D). This might be due to the fact that branched chain amino acid metabolism feeds into β-oxidation and the citric acid cycle and thus competes with fatty acid metabolism (Newgard, 2012). Additionally, BCAAs have been shown to impact on metabolism by modulating PI3K-AKT signaling and its downstream transcriptional regulation, in particular of the transcription factor KLF15 (Kruppel-like factor 15) (Liu et al., 2017).

Taken together, we have shown that amino acids contained in the diet, and thus the composition of dietary proteins, have a major differential impact on the evolution of CKD in rats. Aromatic amino acids (AAAs) and branched chain amino acids (BCAAs) are two groups of essential dietary amino acids that displayed seemingly opposite effects, at least over a period of 5 weeks. The AAAs strongly stimulated the RPF and to a lesser extent GFR, possibly *via* an effect on the production of bioactive molecules. The BCAAs rapidly interfered with renal function, decreasing GFR and stimulating kidney fibrosis, thus increasing CKD progression, presumably *via* their effect on energy metabolism. Our results suggest it could be useful to test the administration of dietary proteins containing low levels of branched chain amino acids to slow down CKD progression, in addition to restricting their total amount (Scialla and Anderson, 2013; Koppe and Fouque, 2019).

## Data Availability Statement

All datasets generated for this study are included in the article/Supplementary Material.

## Ethics Statement

The animal study was reviewed and approved by Kantonales Veterinäramt Zürich.

## Author Contributions

SP and FV contributed to the conception and design of the study. SP, PS, BH, ER, and GP performed the experiments. SP wrote the first draft of the manuscript. GP, SP, and FV wrote sections of the manuscript. All authors contributed to manuscript revision and read and approved the submitted version.

### Conflict of Interest

The authors declare that the research was conducted in the absence of any commercial or financial relationships that could be construed as a potential conflict of interest.

## Footnotes

^1^http://www.ncbi.nlm.nih.gov/tools/primer-blast/
