# Supplementary material for: Differential Impact of Dietary Branched Chain and Aromatic Amino Acids on Chronic Kidney Disease Progression in Rats
Source: Front Physiol. 2019 Dec 9;10:1460. doi: 10.3389/fphys.2019.01460 (PMC6913537; doi:10.3389/fphys.2019.01460)
Supplement: Supplementary file 1 [file Data_Sheet_1.pdf]

Supplementary Material

Differential Impact of Dietary Branched Chain and Aromatic Amino Acids on Chronic Kidney Disease Progression in Rats

Samyuktha Muralidharan Pillai, Brigitte Herzog, Petra Seebeck, Giovanni Pellegrini, Eva Roth and François Verrey

Table S1: GFR measurements on Sham operated animals on different diets

Group	GFR (ml/min/100 g BW) *Mean +/- SEM		
	W4	W6	W9
8+10	1.39 +/- 0.08	1.39 +/- 0.07	1.41 +/- 0.08
CD	1.41 +/- 0.07	1.38 +/- 0.10	1.38 +/- 0.10
BCAA	1.39 +/- 0.10	1.40 +/- 0.08	1.39 +/- 0.09
AAA	1.40 +/- 0.08	1.39 +/- 0.08	1.38 +/- 0.09
EAA	1.38 +/- 0.1	1.40 +/- 0.07	1.42 +/- 0.02
NEAA	1.38 +/- 0.08	1.41 +/- 0.07	1.39 +/- 0.08

*n=5 animals per group

Table S2: RPF measurements on Sham operated animals on different diets

Group	RPF (ml/min/100 g BW) *Mean +/- SEM
8+10	5.52 +/- 0.36
CD	5.48 +/- 0.38
BCAA	5.60 +/- 0.32
AAA	5.51 +/- 0.34
EAA	5.49 +/- 0.36
NEAA	5.50 +/- 0.33

*n=5 animals per group

Table S3: Plasma amino acid levels in 8+10, BCAA and AAA animals with CKD 9 weeks after the second surgery

	8+10		BCAA		AAA	
	*Mean	SEM	*Mean	SEM	*Mean	SEM
Gly	371.0	28.6	384.7	13.7	386.4	8.6
Ala	428.7	16.8	428.7	9.8	449.4	25.0
Val	218.2	17.1	182.3	13.1	209.9	7.2
Leu	179.5	13.2	157.9	11.7	171.3	8.2
Ser	194.8	11.7	199.0	7.0	247.4	10.5
Thr	211.5	23.8	196.3	10.0	264.9	5.8
Pro	186.4	34.3	151.7	6.7	180.3	8.5
Gln	705.6	43.4	693.9	52.5	707.4	20.9
Lys	477.3	25.2	515.1	19.1	467.8	17.5
Ile	114.7	6.4	105.7	8.6	112.1	4.4
Met	53.5	3.0	47.6	2.1	50.1	1.0
Asn	66.3	6.3	62.0	2.8	79.2	4.0
Phe	66.3	4.2	60.7	1.8	49.8	1.4
Tyr	68.3	4.1	64.1	2.4	62.7	3.2
Trp	107.0	12.4	86.4	4.0	79.7	5.1
Arg	134.7	9.2	139.6	4.3	129.2	5.9
His	61.3	4.2	54.2	4.0	55.3	2.2
Asp	7.3	0.6	6.1	0.1	4.1	0.7
Glu	72.4	5.5	69.2	6.8	42.2	2.9

*n=8 animals per group

Table S4: Amino acid composition in casein*

Amino acid	g/16gN
Alanine	2.8
Arginine	3.5
Aspartic Acid	2.6
Asparagine	4.0
Cystine	0.4
Glutamic Acid	8.4
Glutamine	11.4
Glycine	1.7
Histidine	2.9
Isoleucine	5.3
Leucine	9.2
Lysine	7.5
Methionine	2.7
Phenylalanine	4.9
Proline	9.5
Serine	5.7
Tyrosine	5.3
Threonine	4.2
Tryptophan	1.3
Valine	6.7

*adapted from Granovit AG, Switzerland
